# Stone formation in peach fruit exhibits spatial coordination of the lignin and flavonoid pathways and similarity to *Arabidopsis *dehiscence

**DOI:** 10.1186/1741-7007-8-13

**Published:** 2010-02-09

**Authors:** Christopher D Dardick, Ann M Callahan, Remo Chiozzotto, Robert J Schaffer, M Claudia Piagnani, Ralph Scorza

**Affiliations:** 1 Appalachian Fruit Research Station, United States Department of Agriculture, Agricultural Research Service, Kearneysville, WV, 25430, USA; 2 Department of Crop Production, Fruit Tree Unit, University of Milan, Milan 20133, Italy; 3 The New Zealand Institute of Plant and Food Research, Mt Albert, Auckland 1142, New Zealand

## Abstract

**Background:**

Lignification of the fruit endocarp layer occurs in many angiosperms and plays a critical role in seed protection and dispersal. This process has been extensively studied with relationship to pod shatter or dehiscence in *Arabidopsis*. Dehiscence is controlled by a set of transcription factors that define the fruit tissue layers and whether or not they lignify. In contrast, relatively little is known about similar processes in other plants such as stone fruits which contain an extremely hard lignified endocarp or stone surrounding a single seed.

**Results:**

Here we show that lignin deposition in peach initiates near the blossom end within the endocarp layer and proceeds in a distinct spatial-temporal pattern. Microarray studies using a developmental series from young fruits identified a sharp and transient induction of phenylpropanoid, lignin and flavonoid pathway genes concurrent with lignification and subsequent stone hardening. Quantitative polymerase chain reaction studies revealed that specific phenylpropanoid (phenylalanine ammonia-lyase and cinnamate 4-hydroxylase) and lignin (caffeoyl-CoA O-methyltransferase, peroxidase and laccase) pathway genes were induced in the endocarp layer over a 10 day time period, while two lignin genes (*p-*coumarate 3-hydroxylase and cinnamoyl CoA reductase) were co-regulated with flavonoid pathway genes (chalcone synthase, dihydroflavanol 4-reductase, leucoanthocyanidin dioxygen-ase and flavanone-3-hydrosylase) which were mesocarp and exocarp specific. Analysis of other fruit development expression studies revealed that flavonoid pathway induction is conserved in the related Rosaceae species apple while lignin pathway induction is not. The transcription factor expression of peach genes homologous to known endocarp determinant genes in *Arabidopsis *including *SHATTERPROOF*, *SEEDSTCK *and *NAC SECONDARY WALL THICENING PROMOTING FACTOR 1 *were found to be specifically expressed in the endocarp while the negative regulator *FRUITFU*L predominated in exocarp and mesocarp.

**Conclusions:**

Collectively, the data suggests, first, that the process of endocarp determination and differentiation in peach and *Arabidopsis *share common regulators and, secondly, reveals a previously unknown coordination of competing lignin and flavonoid biosynthetic pathways during early fruit development.

## Background

Plants have evolved a wide array of strategies for seed protection and dispersal. Among these, *Prunus *species including cherry (*Prunus cerasus P. avium)*, peach (*P. persica)*, plum (*P. domestica, P. salicina)*, apricot (*P. armeniaca) *and almond *(P. dulcis*) have developed a unique adaptation where the seed is encased by an extremely hard wood-like carapace called the stone. The stone is formed through lignification of the fruit endocarp layer, a feature that defines a broader class of plants called drupes. Mango (*Mangifera indica)*, olive (*Olea europaea)*, coffee (*Coffea spp*.), coconut (*Cocos nucifera)*, blackberries (*Rubus *spp.) and pistachio (*Pistacia vera) *are all examples of drupes highlighting their diversity and agricultural importance.

Ryugo first recognized in the early 1960s [1,2] that peach stones contained lignin. Lignin is a compound unique to plants and has a tremendous economic importance because of its role in tree crops for use in pulp and paper production, in forage crops for digestibility and, more recently, for biofuels. Over the years, most, if not all, of the enzymes in the lignin biosynthetic pathway and a number of potential regulatory points have been identified [3]. Lignin is formed from the phenylpropanoid (PP) pathway, the end products of which are coniferyl and sinapyl alcohols. These lignin monomers serve as the basis for lignification which is the process of producing the lignin polymer via oxidative processes guided by peroxidases and laccases. Radical coupling of the monomers, particularly cross-coupling with the growing polymer, is a combinatorial process that produces the complex lignin polymer [4].

While it may be particularly prominent in *Prunus *stones, lignin deposition within specific fruit tissue layers is a recurring theme in seed protection and dispersal. In some cases, lignification of fruiting structures evolved to protect the seed from disease and stress [5]. For example, lignification of the cuticle and outer integuments of seeds protects them from herbivory and environmental stress [6,7]. Endocarp lignification in *Arabidopsis *has been well studied in relation to dehiscence. Dehiscence serves as a mechanism of seed dispersal in a number of economically important plant species. Lignification of a thin endocarp layer, called enb, provides tension forces that trigger the forcible opening of the seed pod upon drying and mechanical stimulation. Genetic dissection of this process has identified several transcription factors that mediate enb development including the MADS-box genes SHP1, SHP2 and STK, along with the basic helix-loop-helix genes *ALCATRAZ *(ALC) and *INDEHISCENT *(IND) that promote enb differentiation. Negative regulation is accomplished by *FRUITFUL *(FUL) and *REPLUMLESS *(RPL) that define enb boundaries through restriction of *SHATTERPROOF *(SHP), ALC, *SEEDSTIC *(STK) and IND expression [8-10]. While the mechanism for lignin pathway regulation during dehiscence is not fully understood, two NAM, ATAF AND CUC (NAC) class transcription factors, *SECONDARY WALL THICKENING PROMOTING FACTOR (*NST)1 and 3, were recently found to be associated with secondary wall formation within the enb layer [11]. NST1 is also known to regulate secondary wall synthesis in vegetative tissues, suggesting that reproductive tissues utilize a similar, if not the same, lignification pathway [12].

The mechanism of stone hardening in *Prunus *has only been investigated to a limited extent. Only one or two components or enzymes in the composition and formation of the stone tissue have been examined [2,13-15] including two reports of Tani *et al*. [16,17] on the relationship of FUL, STK and SHP to the split-pit (split-stone) phenotype of peach: a phenomenon associated with early ripening. Many basic questions remain unanswered about the biochemical makeup of stone tissue, how it is formed and whether or not stone tissue differentiation is controlled in the same way as enb development in *Arabidopsis*.

Here we set out to determine the developmental and molecular basis for stone formation during early peach fruit development. Results show that numerous genes within the PP and lignin pathways are induced in stone tissues concurrent with the onset of lignin deposition. The flavonoid pathway was also up-regulated in mesocarp and exocarp during this time period and, presumably, competes for PP pathway derived precursors. Analysis of transcription factors point to the conclusion that peach endocarp differentiation may be regulated in the same fashion as the *Arabidopsis *enb layer. Collectively, the gene expression data are presented in context with that of other plant species and are consistent with known aspects of stone fruit physiology and development.

## Results

### Spatial/temporal pattern of lignin deposition in the peach endocarp

**Figure 1:**
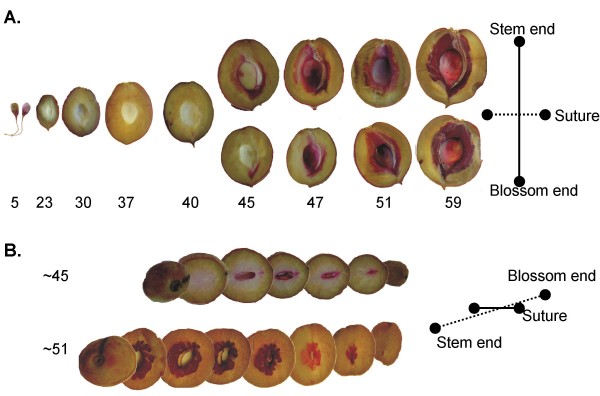
**Progression of lignin deposition in developing peach fruit**. Sectioned fruit were stained with phloroglucinol-HCl for lignin (red colour). Numbers indicate days after bloom (DAB) (A) Fruit series cut perpendicular to the fruit suture. (B) Whole fruit serially cut parallel to the suture at 45 and 51 DAB. The crosses indicate the orientation of the blossom end, the stem end and the suture.

In order to identify the critical stone developmental times during the growth of young fruit, the timing and pattern of lignin deposition was studied. Stone tissue hardens at the transition from stage I to stage II of growth [18,19], therefore fruit were collected at times spanning that transition and stained to detect lignin (Figure 1). In fruit dissected parallel to the suture (the margin running from stem to blossom formed by ovary concatenation), lignin deposition was first detected at 37 days after bloom (DAB) in a thin tissue layer radiating from the blossom end. After that staining rapidly progressed throughout the entire endocarp layer (Figure 1A). The stones began to substantially harden by 59 DAB after which time they could no longer be cut with a knife. Little or no staining was observed in tissues other than the endocarp with the exception of scattered small vascular elements in the mesocarp and trichomes present on the fruit surface. Fruit were dissected perpendicular to the suture at two time points (45 DAB and 51 DAB), which allowed the visualization of lignin deposition starting from the suture side and the formation of the irregular 'flames' characteristic of peach stones (Figure 1B). These data define the general pattern of lignin deposition and demonstrate that the stone lignification process initiates relatively early in fruit development.

### Expression profiling of early peach fruit development

In order to identify genes whose expression patterns correlated with the lignin staining, a time course expression profiling study was performed on seven stages of developing fruit spanning the stone hardening process. Two microarray platforms were used: a small (4806 features) long oligo peach array that was custom printed based on Trainotti *et al*. [20] and a more comprehensive apple oligoarray (15,000 features) [21] which was used because both apple and peach are in the Rosaceae family and share a high degree of nucleotide identity. Labelled cDNA samples from four or seven time points were hybridized to the peach array and apple array, respectively (Additional File 1). All hybridizations used reference cDNA derived from 87 DAB peach fruit minus the stone; a stage where the fruit had not begun to ripen but stone hardening was complete. This time point was chosen as the mesocarp and exocarp tissues do not undergo lignification and, therefore, would emphasize stone-related gene expression.

Nine hundred and seven genes were identified from the peach array hybridizations and 5546 genes from the apple array hybridizations that showed statistically significant expression changes (Additional File 2). Additional analyses were done in order to verify the validity of the apple array data as it had been derived from a related yet distinct species. Cross-species hybridizations have been routinely used for other plant families such as Solanaceae and are informative when appropriate data analysis and confirmation methods are used [22-24].

Twenty-five per cent of the peach genes that displayed statistically significant changes in gene expression (227) were also identified as differentially expressed in experiments using the apple array based on blastN *e*-value >10^-20^. This percentage is slightly lower than the estimated per cent overlap of total gene content present on the two array platforms (data not shown). More than 75% of the shared significant genes showed identical, or highly similar, expression patterns, confirming the assumption that the apple arrays yielded informative results. Expression graphs for a random sample of 25 genes are shown in Additional File 3.

Recently, an unpublished draft of the peach genome sequence has become available allowing us to predict which apple array oligos would be likely to hybridize to their intended targets based on BlastN analysis. The results showed that approximately 80% of all apple array oligos share a significant homology with a peach target (Additional File 2) which is consistent with the results of our array data comparisons. Genes represented by oligos that gave poor BlastN scores (<17 bp contiguous match) were flagged in the final data set (Additional File 2) but not eliminated. These data were included as a low BlastN score does not invalidate them because some oligos predictably span introns (which would interrupt BlastN alignments) and the cutoff score used is potentially above the actual hybridization threshold which is influenced by the abundance of the target messenger RNA, the abundance of competing non-target RNAs, and the T_m_. In fact, one of the apple array significant genes (peroxidase; POX), that we later confirmed by quantitative polymerase chain reaction (qPCR), was represented by an oligo that only had a 16 bp contiguous match with its intended peach target (Additional File 2).

**Figure 2:**
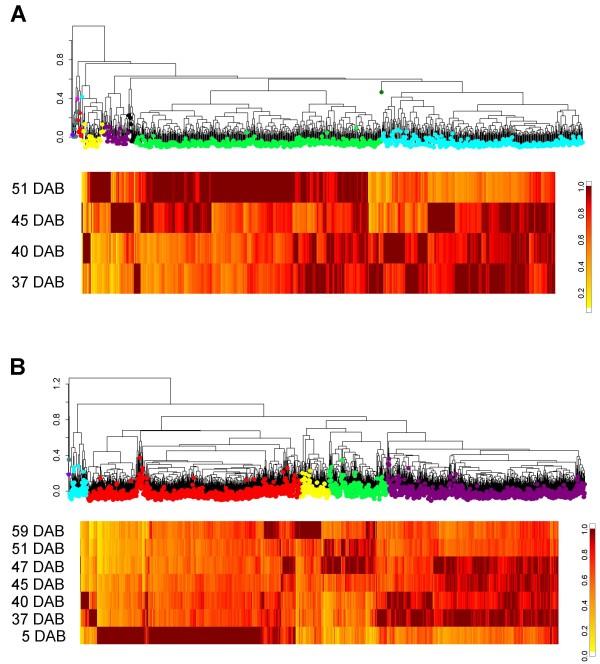
**Hierarchical cluster of 907 selected genes from the μPeach1.0 microarray (A) and 2548 selected genes from the 15 K apple microarray (B)**. Each gene was scaled to 1 to represent maximum expression to allow cluster separation by expression pattern. The colours for each of the groupings were chosen to differentiate the groups and do not relate to the peach and apple groupings. The yellow to red scale to the right of each figure is a colour scale representing the expression level of each gene as it relates to the highest time point of expression. The intense red represents the maximum time of expression and the yellow represents the lowest per cent of that maximum expression. As the genes are different in each of the array platforms, these scales can not be compared between the platforms.

The selected genes from each microarray were grouped according to expression pattern by cluster analysis. The most notable pattern from this analysis was a large group of expressed genes that were at their highest and lowest expression from the 5 DAB sample on the apple array or on the 51 DAB sample on the peach array. The 5 DAB may be influenced by seed specific expression since seeds were removed at all subsequent time points (Figure 2). The 51 DAB sample is the only sample used on the peach array where lignification was readily detected.

Expression data from the combined peach and apple array datasets was analysed for genes which had expression patterns consistent with the timing of lignin deposition. First, figure of merit (FOM) analysis was performed to determine the number of clusters needed to explain the majority of variation in expression patterns [25]. Next, this value (12) was used as the input parameter for K-means clustering (KMC) to divide the data into twelve distinct expression clusters [26] (Additional File 4). One cluster (No. 8) contained genes with expression patterns similar to the lignin deposition pattern derived from phloroglucinol-HCl staining. The most highly induced genes within this cluster fell into three major metabolic pathways; flavonoid biosynthesis, lignin biosynthesis and the phenylpropanoid pathway (PP) which produces the precursors for both flavonoid and lignin biosynthesis.

**Figure 3:**
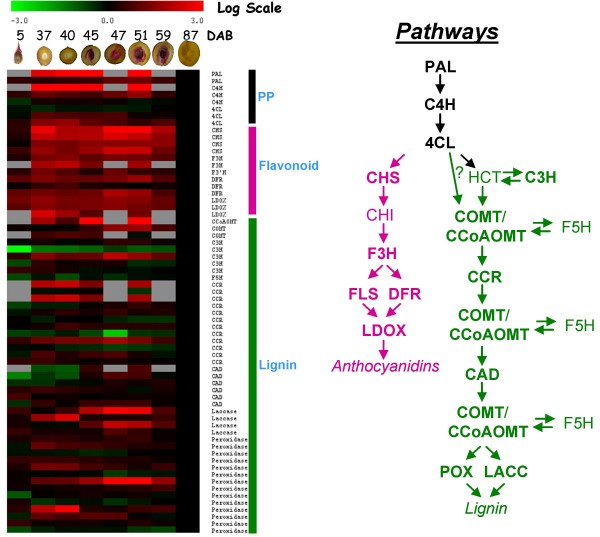
**Induction of the phenylpropanoid pathway (PP), lignin and flavonoid pathways during fruit development**. A heat map is shown for all significant PP, lignin and flavonoid pathway genes from the combined peach and apple microarray data. Log_2-_fold expression scale is shown at top. Developmental times are indicated as days after bloom along with a fruit image stained for lignin deposition. Gene abbreviations are listed along with colour coded bars indicating the corresponding pathway. A sketch of the PP, lignin and flavonoid pathways is shown on the right. Names for induced genes as determined from the array data are shown in bold.

The expression patterns for all the significant genes (as defined previously by statistically significant expression changes during early fruit development) in those three pathways were compared in the form of a 'heat' map (Figure 3). Many, but not all, of those genes had expression profiles similar to the lignin deposition profile which was partially defined by maximum expression between 45 and 51 DAB, consistent with the observed proliferation of lignin staining.

### Validation of array data via qPCR

**Figure 4:**
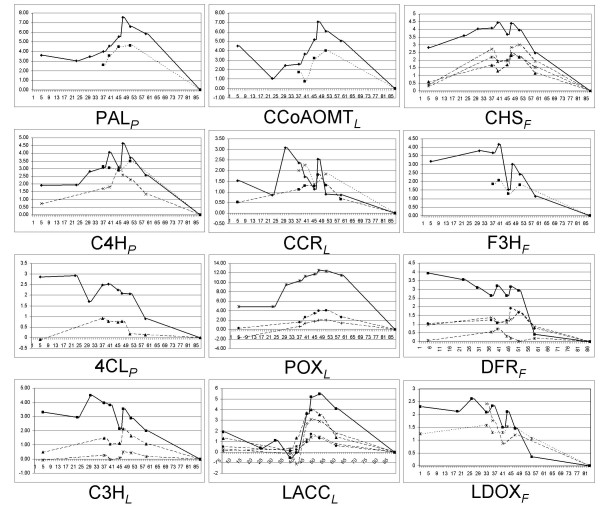
**Validation of array data using quantitative PCR (qPCR)**. Gene abbreviations are shown below each graph. Corresponding pathway is indicated after each abbreviation as phenylpropanoid _*P*_, lignin _*L *_or flavonoid _*F*_. Y-axis represents Log_2_-fold change relative to values (>0) obtained from the 87 days after bloom (DAB) reference sample. *X*-axis is DAB. Some of the genes were present only in the peach array, some only in the apple array (4-coumarate CoA ligase) some in both (cinnamoyl CoA reductase) and some are represented by multiple oligos (chalcone synthase). Data from the qPCR is shown as solid lines, apple array data as dotted lines and peach array data as dashed lines.

From the significant gene list (Additional File 2), 12 genes showing high homology to known secondary metabolism genes were chosen for further validation by qPCR. These included three PP, five lignin and four flavonoid pathway genes (Additional File 5). cDNA derived from two additional fruit developmental time points (23 and 30 DAB) were included in order to get a more detailed expression pattern for these genes. The relative expression was calculated as the Log2- fold change relative to values obtained from the qPCR for the 87 DAB reference sample to enable direct comparison to the array data. The data derived from the peach and apple array platforms were in close agreement with the qPCR results based on the overall expression pattern (Figure 4). Some variation was observed for specific time points and, in the case of cinnamoyl CoA reductase (CCR), expression was highest at 30 DAB which was a time point not included in the array experiments. One source of variation could owe to the fact that many of these genes are members of gene families and it is likely that the array data represents the combined signal of more than one gene. Upon preliminary release of the peach genome we checked to see if our qPCR primers were specific to a single gene or could potentially amplify more than one gene family member using BlastN searches. All qPCR primer sets were specific to a single gene with the exception of *p-*coumarate 3-hydroxylase (C3H) which was found to be encoded by several nearly identical copies present in tandem (data not shown).

Next, we plotted the normalized absolute expression data (as opposed to Log_2-_fold change shown in Figure 4) on a linear time scale and found two or more reoccurring expression patterns among these genes (Additional File 6). Phenylalanine ammonia-lyase (PAL), cinnamate 4-hydroxylase (C4H), caffeoyl-CoA O-methyltransferase (CCoAOMT), POX and a laccase (LACC) all showed a strong and rapid increase in gene expression that initiated at 40 DAB and peaked at 47 DAB. In contrast, the PP gene 4-coumarate CoA ligase, the lignin pathway genes C3H and CCR and the flavonoid pathway genes chalcone synthase (CHS), leucoanthocyanidin dioxygen-ase (LDOX), dihydroflavanol 4-reductase (DFR), and flavanone-3-hydroxylase (F3H) showed overlapping expression patterns with periodic induction at two or three time points (30, 40 and 47 DAB).

### Spatial expression of lignin and flavonoid pathway genes throughout the fruit

In order to determine if there was a spatial regulation as well as temporal, fruit were collected the following year (2007) at the peak lignin gene induction time point (47 DAB) and dissected into endocarp, mesocarp, exocarp and seed. The total RNA was extracted from each tissue and used for qPCR. Eleven representative genes were tested in order to determine if their expression was tissue specific. Results showed that the two PP genes (PAL and C4H) and three lignin genes (CCoAOMT, POX and LACC) that had a single peak induction time at 47 DAB in whole fruit were largely specific to the endocarp, while the lignin pathway genes CCR and C3H and flavonoid pathway genes CHS, DFR, LDOX and F3H were expressed similarly in all four fruit tissues (Additional File 7). In order to place the observed lignin genes induction levels in context of other lignifying tissues, we also tested PAL, C4H, CCoAOMT and POX expression in developing peach wood (Additional File 8). Expression in wood was not significantly different from reference fruit (87 DAB) with stone expression being 30-40 times higher than wood for PAL, C4H and CCoAOMT and over 5000-fold higher for POX.

**Figure 5:**
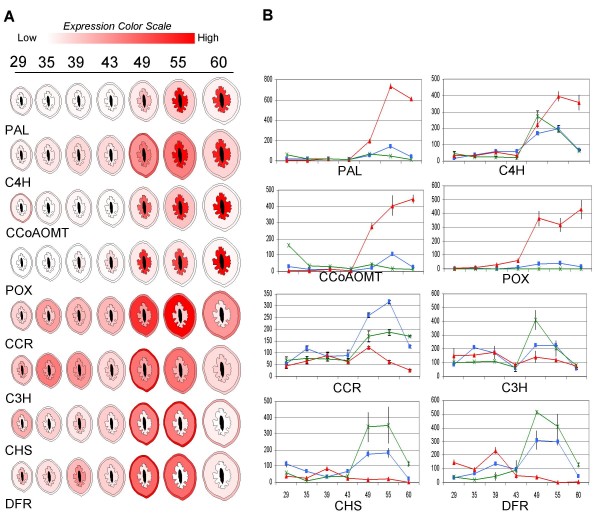
**Spatial/temporal expression of eight phenylpropanoid, lignin and flavonoid pathway genes during fruit development**. Gene abbreviations are indicated beneath an artificial rendering of the dissected fruit development series. Outer section represents exocarp (skin), middle section is mesocarp (flesh) and inner portion is endocarp (stone). No expression data was obtained for the seed which is represented in black. Fruit collection times are shown at top as days after bloom (DAB). Normalized relative expression values are indicated by a sliding colour scale. (A) Highest expression levels are shown in red while lowest expression values are white. (B) Actual relative expression values are graphed for each tissue section: endocarp (red), mesocarp (blue), exocarp (green). *Y*-axis is relative expression value based on a standard dilution curve. *X*-axis values are DAB.

The following year (2008), we collected an additional fruit developmental series, beginning before the onset of lignin deposition and ending at maximum lignin deposition. All fruit were dissected into endocarp, mesocarp and exocarp. In 2008, lignification occurred about 10 days later than in 2006 and 2007, probably due to the long cool spring, but the fruit were at similar developmental stages based on phloroglucinol-HCl staining (data not shown). qPCR studies were performed on all sectioned tissues and time points for 8 representative genes (PAL, C4H, CCoAOMT, POX, CCR, C3H, CHS and DFR). Results are summarized in Figure 5. PAL, CCoAOMT and POX showed a marked increase in expression in the endocarp with relatively little expression in the mesocarp or exocarp, confirming previous data. We can not rule out the possibility that the small levels of induction observed in the mesocarp were due to imprecise dissection of the endocarp and mesocarp which have an ungulate, non-uniform boundary. C4H showed marked induction in the endocarp but was also substantially induced in both the mesocarp and exocarp layers peaking about 1 week earlier. This result is consistent with the additional minor peaks observed in the 2006 whole fruit data sets. Unlike lignin pathway genes, the expression of the flavonoid genes CHS and DFR was predominantly in the mesocarp and exocarp. Induction initiated around the same time as PP and lignin pathway genes but a smaller peak was also observed in the endocarp before lignin deposition or lignin gene induction. The lignin pathway genes CCR and C3H showed overlapping expression patterns with CHS and DFR, the differences being that C3H was not substantially induced in the flesh while CCR showed a slight peak in the endocarp at 49 DAB. As C3H qPCR primers were not specific to a single gene we can not rule out the possibility that individual C3H family members are differentially expressed in these tissues. It is also interesting to note that CHS and DFR expression in the endocarp was lowest when lignin pathway genes were highest. Collectively, these data suggest that the periodic induction patterns observed for flavonoid pathway genes, as well as CCR and C3H, in the 2006 whole fruit studies are probably due to separate induction events in the endocarp, mesocarp and exocarp.

### Identification of fruit lignin and flavonoid pathway regulons

Given the apparent physiological significance of the distinct, yet overlapping, lignin and flavonoid gene expression patterns, we next attempted to identify corresponding regulons from the microarray data via Pavlidis template matching (PTM) using the validated genes as templates [27]. Results are shown in Additional File 9. Using a cluster formed from PAL, CCoAOMT, POX and LACC as a template, 290 genes (regulon 1) were identified (*P*-value 0.01) showing the stone specific expression pattern. When using the flavonoid genes CHS, DFR, F3H and LDOX, 208 genes (regulon 2) showed a matching expression pattern (*P*-value 0.01). The two datasets overlapped by 18 genes and among them was C4H. As expected, lignin pathway genes were predominantly found within regulon 1 while flavonoid genes were abundant in regulon 2. Careful manual classification of genes in both regulons revealed that they contained similar classes of genes with a few exceptions (Table 1). Regulon 1 was enriched in cell wall synthesis/modifying genes including cellulose synthases, phytochelatin synthases and polygalacturonases. Regulon 1 also contained a number of genes encoding glycolytic enzymes that were absent in regulon 2, while regulon 2 was enriched for starch biosynthesis genes.

**Table 1:** Categorization of genes represented in lignin and flavonoid regulons.

Category/subcategory	Regulon 1	Regulon 2	InvRegulon 1	InvRegulon 2
Amino acid metabolism	8	7	4	16
Cell cycle	2	1	0	2
Cell wall	20	6	6	14
Chloroplast/light response/photosynthesis	3	1	5	14
Development	17	12	10	23
DNA binding/histone/chromatin folding/repair	5	1	3	5
Energy/mitochondria	5	2	4	12
General metabolism/catabolism	10	11	8	35
Biosynthesis: starch	0	5	1	9
Glycolysis	5	0	2	9
Lipid/fatty acid metabolism	3	5	4	9
Membrane/cytoskeleton/intracellular transport	11	7	4	9
Nucleic acid/nucleotide sugars metabolism	3	2	2	4
Oxidation/reduction	5	2	2	12
Protein synthesis/ribosome/transfer RNA/chaperone	7	1	16	15
Proteolysis/protease/proteasome	9	12	12	23
RNA binding/synthesis/modification	4	6	6	11
Secondary metabolism	18	23	4	4
Biosynthesis: flavonoid	2	13	3	3
Biosynthesis: lignin	11	5	1	1
Biosynthesis: phenylpropanoid	5	4	0	0
Stress/pathogenesis	4	6	6	21
Transporter/pump/ion channel	9	3	10	28
Unknown	136	90	81	167
Vacuole function	3	0	1	12
*Total genes*	*282*	*198*	*189*	*440*

Inv = Inverse

PTM was also performed for genes showing the polar opposite expression patterns as regulons 1 and 2; in other words genes repressed during lignin or flavonoid pathway induction (Table 1). Genes expressed inversely to regulons 1 and 2 included those involved in protein synthesis as well as a various membrane transporters. Surprisingly, a few lignin genes were also present in inverse regulon 1 including CCR, catechol-O-methyl transferase and LACC family members.

### Comparison to other fruit expression studies

We subsequently analysed data from existing apple, tomato and pepper fruit expression profiling studies in order to determine if the observed induction of PP, lignin and flavonoid pathway genes were species specific [28-30] (Additional Files 10 and 11). The flavonoid pathway genes CHS, F3H, DFR and LDOX were up-regulated in early apple fruit development but not substantially induced in pepper or tomato. In contrast, the lignin pathway was not induced during apple or tomato fruit development but was steadily induced during pepper ripening. This is consistent with the apparent role of the lignin pathway in capsaicinoid biosynthesis [31].

### Expression of transcription factors during lignin deposition

Next we attempted to identify regulatory factors that potentially control regulons 1 and 2. Candidate transcription factors were first chosen from the microarray data. We initially targeted NAC transcription factors which are known to control secondary wall formation in woody tissues and MYBs which are known to control flavonoid biosynthesis in other fruits [12,32]. Robust qPCR data was obtained for two candidate NACs (one each from regulons 1 and 2) and three MYBs that showed substantial expression changes but were not identified as members of either regulon. The regulon 1 NAC gene [GenBank:EB155211] was consistent with the regulon 1 pattern overall but was found to be mesocarp and exocarp specific (data not shown.). The remaining four transcription factors [GenBank:EB131006, GenBank:EG631309, GenBank:CN908525 and GenBank:CN139017] showed flesh and skin specific patterns consistent with the onset of flavonoid pathway induction (Additional File 12.). Together, these three MYBs and the one NAC are candidates for flavonoid pathway regulation, but no candidates were identified as potential lignin regulatory factors.

The process of dehiscence in *Arabidopsis *is among the best characterized examples of endocarp development and lignification. Thus, for comparative purposes, putative homologues of *Arabidopsis *genes known to control enb layer development and lignification were studied. Peach homologues of SHP, STK, and FUL were previously identified by Tani *et al*. [16,17]. From the recently completed draft peach genome, we were able to identify putative homologues of NST1, ALC and IND via BlastX searches (D Main and B Sosinski, personal communication). Additional TblastN searches revealed that none of these genes were represented on either the apple or peach oligoarrays (data not shown).

**Figure 6:**
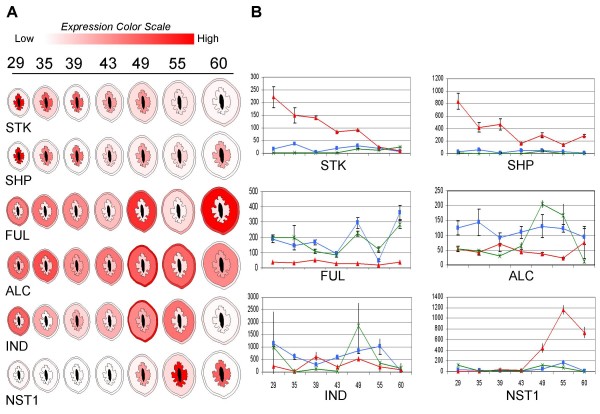
**Spatial/temporal expression of peach homologues of known dehiscence regulatory factors during fruit development**. Gene abbreviations are indicated beneath an artificial rendering of the dissected fruit development series. Outer section represents exocarp (skin), middle section is mesocarp (flesh) and inner portion is endocarp (stone). No expression data was obtained for the seed which is represented in black. Fruit collection times are shown at top as days after bloom (DAB). Normalized relative expression values are indicated by a sliding colour scale. (A) Highest expression levels are shown in red while lowest expression values are white. (B) Actual relative expression values are graphed for each tissue section: endocarp (red), mesocarp (blue) and exocarp (green). *Y*-axis is relative expression value based on a standard dilution curve. *X*-axis values are DAB.

We performed qPCR studies using the 2008 dissected fruit series for SHP, STK, FUL, ALC, IND and NST1 (Figure 6). Both SHP and STK were found to be endocarp specific, showing peak expression at 29 DAB (the earliest stage fruit) and gradually declining to a minimum near the onset of lignin pathway induction. SHP expression was found to persist in the endocarp (albeit at a lower level) throughout lignification while STK did not. ALC and IND did not show substantial expression changes or tissue specificity, though it is worth noting that IND expression declined in all tissues at 60 DAB. FUL expression remained relatively low in the endocarp throughout the developmental series. In contrast, NST1 expression initiated at the same time as lignin deposition and showed an identical expression pattern as the PP and lignin pathway genes PAL, CCoAOMT and POX.

## Discussion

Endocarp lignification plays critical roles in seed protection and dispersal in some fruits and yet it occurs sporadically throughout angiosperm lineages. This prompts the question of whether it is an ancestral state of angiosperms or a more recent adaptation. Among plants in the family Rosaceae, *Prunus *is one of two genera (the other being *Rubus*) that form a lignified endocarp layer which provides an excellent opportunity to address evolutionary questions. Here we sought to better characterize peach stone formation and define the molecular pathways that control it in order to gain an insight into how *Prunus *species evolved a lignified endocarp. Results show that the peach endocarp layer accumulates lignin 5-6 weeks after bloom. Lignin deposition proceeds from the blossom end and extends throughout the entire endocarp over a ten day time period (Figure 1). Recent biochemical studies have shown that peach stones accumulate extremely high lignin contents (≈ 50% lignin) relative to other woody tissues (≈ 25% lignin) (R Scorza, J Ralph and F Lu, unpublished data). Therefore, understanding how peach stones accumulate so much lignin could have important implications for forestry, forage and bioenergy crops in which lignin regulation is central to a number of critical agricultural traits.

Global gene expression analysis during peach fruit development revealed the up-regulation of a number of PP, lignin and flavonoid pathway genes concurrent with lignin deposition and stone hardening (Figure 3). Genes in these pathways made up over 20% (14/65) of annotated genes showing >3 Log_2-_fold expression. The concurrent induction of the lignin and flavonoid pathways is in sharp contrast since these are competitive pathways that presumably draw on the same precursors generated by the PP pathway. Expression studies in dissected fruit revealed that there is a distinct spatial separation of some components of the two pathways (Figure 5). The PP gene PAL, which catalyzes the first step in PP biosynthesis, and three lignin pathway genes (CCoAOMT, POX and LACC) were found to be largely endocarp specific while expression of the flavonoid genes (CHS, DFR, F3H and LDOX) and two lignin pathway genes (C3H and CCR) were predominately expressed in the mesocarp and exocarp. C4H, which catalyzes the second step in the PP pathway, showed expression throughout the fruit but transcripts predominated in the endocarp (Figure 5, Additional File 7). The overlap in expression of the known lignin pathway genes C3H and CCR with the flavonoid pathway implies that they may have flavonoid associated functions. In other plant species, CCR and C3H genes tend to be comprised by small gene families and have probably diverged [33]. Our gene expression data suggests that that the identified C3H and CCR family members may not be rate limiting to lignin biosynthesis but may play important roles in flavonoid metabolism. While these inconsistencies have yet to be resolved, collectively, the expression data reveals intricate connections between lignin and flavonoid pathway regulation during peach fruit development.

The identified lignin and flavonoid regulons (1 and 2, respectively) reveal additional cellular changes associated with secondary metabolism in fruits (Additional File 9). Not surprisingly, regulon 1 includes a number of cell wall biosynthesis and secondary wall formation enzymes. Cell wall modifications are essential for proper lignin polymerization and hardening [34]. The shift to increased secondary metabolism also appears to be associated with decreased protein synthesis and membrane transporter expression. These changes may reflect cellular metabolic rewiring necessary to enable extreme increases in secondary metabolism.

The observed spatial/temporal coordination between lignin and flavonoid expression supports the model that lignin and flavonoid biosynthesis are competitive. During times of peak lignin deposition genes in the lignin biosynthesis pathway were strongly induced while flavonoid pathway genes were repressed (Figure 5). Expression levels of CHS and DFR were lowest in lignifying stone tissue relative to other developmental times or during ripening. Conversely, high flavonoid gene expression was correlated with lower expression of genes involved in lignin biosynthesis. This interpretation is complicated by our finding that PP pathway genes, PAL and C4H, were disproportionally associated with lignin pathway induction. Little PAL expression was observed in the flesh or skin even when flavonoid gene expression was at its peak. In contrast, C4H showed substantial induction in the mesocarp and exocarp, though still to a slightly more limited extent than the endocarp. This discrepancy could be explained by the fact that PAL is typically encoded by two to four closely-related genes while C4H is often a single gene [33]. An initial survey of lignin and flavonoid gene families in the draft peach genome suggests that there may only be two PAL genes and a single C4H, while other PP and lignin pathway gene families appear to be similar in size as *Arabidopsis *(data not shown). Thus, we interpret the data to mean that unidentified PP family members may function in the mesocarp and exocarp, that PP precursors for flavonoid biosynthesis are produced at sufficient but relatively lower PP gene expression levels and/or that the flavonoid pathway can be fed by an, as of yet unidentified, pathway in fruit tissues. In previous functional studies, silencing of individual PP genes in plants has shown marked decreases in lignin biosynthesis with more limited impacts on flavonoid production [35,36]. As with the current study, these apparent inconsistencies have gone largely unexplained but collectively point to the conclusion that at least some enzymes in the PP pathway may not be rate limiting to flavonoid biosynthesis. Upon public release of the peach genome sequence (currently being assembled, D Main and B Sosinski, personal communication), it should be possible to differentiate each family member and confirm whether or not the PP pathway is substantially up-regulated during flavonoid biosynthesis.

Mining of gene expression databases for apple, tomato and pepper revealed that induction of the lignin pathway in young fruit is unique to *Prunus*, while flavonoid pathway induction may have a more ancient origin. The lack of obvious flavonoid induction in pepper and tomato is consistent with the lack of anthocyanins in these fruit which derive their red colour primarily from carotenoids. In contrast, the induction of the flavonoid pathway in anthocyanin rich fleshy fruits is supported by studies in both strawberry and grape [37,38]. In addition to colour, the flavonoid pathway contributes to a number of important agricultural traits including flavour, nutritive properties and disease/stress resistance. The combined data from peach and apple fruit development studies indicates that the early induction of the flavonoid pathway is limited to genes encoding enzymes involved in the initial steps of flavonoid biosynthesis and proanthocyanidin production.

When placed in a physiological context, the expression patterns of lignin and flavoniod pathway genes are consistent with known aspects of peach fruit development. Peach fruit grow on a sigmoidal curve and show a growth plateau that coincides with the timing of stone hardening. Previous studies in plum fruit show that stones rapidly begin to accumulate dry weight during this time period [39]. This slow down in fruit expansion could be attributed to the substantial energy resources which go in to endocarp lignification and hardening. Our data support this model as lignin gene expression is induced at extremely high levels immediately prior to the slow down in fruit growth. What is perhaps surprising is that expression of flavonoid biosynthesis genes in the flesh and skin appears to occur around the same time as the onset of lignification but diminish before the endocarp substantially hardens. Thus, energy resources in the fruit appear to be carefully partitioned to enable flavonoid accumulation before stone hardening depletes the necessary energy and metabolic resources. Here, the peach cultivar 'Suncrest' was used which is a yellow fleshed variety with red skin. This colour pattern mirrors the higher flavonoid gene expression that we observed in skin. Thus, other peach cultivars with different colour patterns, such as red flesh or yellow skin, may have different flavonoid gene expression patterns. However, flavonoid gene induction is not necessarily associated with anthocyanin production especially since 'Suncrest' has yellow and not red flesh. Rather, it seems likely that early flavonoid induction may also function to protect young fruit against disease and herbivory. Both the lignin and flavonoid pathways are induced during stress and pathogen attack and function to enhance tissue rigidity, decrease digestibility and produce anti-microbial compounds [40]. Young fruit tend to be highly resistant to pathogens and are undesirable to herbivores, in part, due to the presence of flavonoid compounds [41,42]. *Prunus *fruits tend to become more susceptible to pathogens after stone hardening and become attractive to herbivores during ripening [43-45]. Thus, the flavonoid pathway serves somewhat opposite functions in *Prunus *fruits; pathogen and herbivore resistance in young fruit and herbivore attraction when fruit are mature and seeds are ready for dispersal. Still, it is important to bear in mind that the roles of the lignin and flavonoid pathways in fruit do vary substantially, as highlighted by pepper where lignin pathway induction during later stages of ripening drives capsaicinoid production which confers herbivore specificity [31,46].

Endocarp lignification occurs in a wide range of angiosperms, including both dry and fleshy fruits. This implies that it is either an adaptive process that occurs through relatively simple evolutionary changes or that it represents an ancestral state in which case fruits with non-lignifying endocarps would have intermittently lost this character. In order to address this question, we examined the expression patterns of peach homologues of *Arabidopsis *genes known to control dehiscence. In *Arabidopsis*, SHP1/2, STK, IND and ALC act together to define the enb layer boundary and are under negative regulation by FUL and RPL [10]. A previous expression study of SHP, STK and FUL, in peach fruit dissected 30 days after full anthesis, found that SHP was endocarp specific, STK was higher in mesocarp and FUL was substantially expressed in both the endocarp and mesocarp. This indicates differences in the control of peach stone formation and *Arabidopsis *dehiscence [17]. We found that both SHP and STK were endocarp specific and steadily declined from the earliest fruit stage analysed (29 DAB) while FUL was consistently lower in the endocarp than the mesocarp or exocarp (Figure 6). These patterns mirror those found for the *Arabidopsis *counterparts and are consistent with a putative role for FUL as a negative regulator of SHP and STK [47]. It is worth noting that FUL expression did not increase in the endocarp as SHP and STK declined. Thus, it appears that SHP and STK are not actively regulated by dynamic FUL levels in the endocarp; rather, it is probably the relative ratio of FUL that enables SHP and STK to promote endocarp differentiation. Surprisingly, ALC and IND expression did not significantly vary with respect to tissue type or developmental time. However, we can not rule out an endocarp specific role as these genes potentially act much earlier in fruit development than analysed here. In *Arabidopsis*, NST1 promotes enb lignification after tissue identity has been established [11]. The decline of SHP and STK expression just prior to the onset of lignin deposition, followed by subsequent induction of NST1, suggests this same regulatory process may occur in peach stones. Collectively, these data indicate that peach stone formation and *Arabidopsis *dehiscence appear to be controlled by a highly conserved pathway of positive and negative regulatory transcription factors that first establish tissue identity and then, subsequently, activates a common pathway in order to promote secondary wall formation and lignification. These close similarities imply that endocarp lignification is probably an ancestral state of angiosperm fruit development. It is an intriguing possibility that the concomitant flavonoid pathway induction observed in fleshy fruit mesocarp and exocarp layers may also be more widely conserved and is, likewise, an ancestral condition.

## Conclusions

Endocarp lignification in peach occurs in concert with a separate induction of the competing flavonoid pathway in the mesocarp and exocarp tissue layers. Flavonoid induction appears to be conserved among Rosaceae species, and possibly, many other fleshy fruits, while lignin pathway induction is not. The coordination of these two processes is likely to be critical for the control of a number of important fruit and nut agronomic characters. Furthermore, both peach and *Arabidopsis *endocarp development seem to be controlled by very similar mechanisms that include the regulatory transcription factors SHP and STK (which promote endocarp differentiation), FUL (a negative regulator) and NST1/3 that trigger secondary wall formation and lignin deposition.

## Methods

### Fruit collection and lignin staining

Three neighboring trees of Suncrest were marked for collection. Bloom time was noted when 50% of flowers had opened. Fruit was collected at 5, 23, 30, 37, 40, 45, 47, 51 and 59 DAB for the 2006 collection. At each collection time, 10 fruit were collected from each tree and the length measured. Five of these were frozen in liquid N2, lyophilized for 6 days and stored at -20°C for future RNA extractions. The remaining five were sectioned and placed immediately in phlorogucinol-HCl staining solution [5% phloroglucinol, 85% ethanol (v/v)], drained and exposed to 100% HCl. The fruit was then rinsed in 95% ethanol (v/v) and photographed. In 2007, fruit was collected at 47 DAB and approximately 10 from each tree were dissected into skin, flesh, stone and seed, frozen in liquid N2, lyophilized for 6 days and stored at -20°C for future RNA extractions. In 2008 fruit was collected at 29, 35, 39, 43, 49, 55 and 60 DAB. Five were sectioned and stained with phloroglucinol [1% (w/v) phloroglucinol, 12% HCl (v/v), 85% ethanol (v/v)] for 1 h [48] and five to 10 were dissected and frozen as in 2007. The 87 DAB peach sample was from a collection made in 1987. This sampling time was repeated in 2008 to stain and photograph only.

### RNA purification and labelling

Lyophilized fruit (0.5 g for early stages, 1 g for middle stages and 1.5 g for later stages) was ground in liquid N2 using mortar and pestle to obtain a fine powder. Total RNA was extracted following the protocol from Callahan *et al*. [49]. RNA was DNase 1 treated using Turbo DNAfree™ Kit (Ambion, TX, USA) following the manufacturer's protocol. The RNA was quantified using the NanoDrop nd-1000 (Thermo Scientific, MA, USA) and the quality checked using the Bioanalyzer 2100 (Agilent, CA, USA) according to manufacturer's directions.

RNA was labelled with either AlexaFluor555 or AlexaFluor647 (Invitrogen, CA, USA) using the SuperScript™ Plus Indirect cDNA Labeling System (Invitrogen) according to the manufacturer's protocol. 20 μg of total RNA was used for the RT reaction and for the purification of both the First-Strand cDNA and Fluorescently Labeled cDNA, the CyScribe GFX Purification Kit (GE Healthcare, NJ, USA) was used instead of Invitrogen's Purification Module.

### Peach microarray fabrication

A set of 4806 peach oligonucleotides (70 mers) plus a set of 24 controls were purchased from Operon Biotechnologies Inc (AL, USA; Array-Ready Oligo Set™, Peach Version 1). The oligos were suspended in 1× Nexterion Spot solution (Schott North America Inc, NY, USA) to a final concentration of 600 ng/μl. Microarrays were printed by the University of California, Davis ArrayCore Facility (CA, USA). The oligos were printed on amino coated glass slides, Nexterion Slide A+ (Schott) using a Lucidea Array Spotter (GE Healthcare, NJ, USA). The slides were then baked at 80°C for 2 h to immobilize the oligos and the microarrays were double-sealed under argon gas.

### Peach/apple microarray hybridizations

For microarray studies, each time point was represented by three biological replicates analysed in a dye swap design (six hybridizations per time point). A total of 50 pmol of incorporated dye with at least a FOI of 2.0 (calculated using Base:Dye Ratio Calculator from Invitrogen^® ^[50]) was used for each sample cDNA and the reference cDNA in the hybridization of both microarray platforms. For the peach microarray the buffer and washes were from the Pronto!™ Universal Microarray Kit (Corning, NY, USA) and performed in accordance to the manufacturer's protocol. For the apple microarrays, an automated slide processor (Lucidea - GE Healthcare) was used, with the same conditions described in Schaffer *et al*. [21].

### Microarray data analysis

Dual channel array images were acquired on a GenePix 4000B microarray scanner and analysed with GenePix Pro software (Axon Instruments, CA, USA). Spots were screened visually in order to identify those of low quality. For the statistical analysis and normalization of the expression data, the LIMMA package for the R programming environment was used [51]. Background correction was performed by using the 'normexp' method [52]. Normexp adjusts the local median background, thus avoiding problems with estimates greater than foreground values, and ensures that there are no missing or negative corrected intensities. This strategy of background correction was used in order to avoid an exaggerated variability of log-ratios for low-intensity spots and an offset of 25 was used for both channels in order to further reduce this variability. The resulting log-ratios were normalized by using the global loess method [53].

### Assessment of differential expression

The data was analysed by applying linear model methods [54]. Each probe was tested for changes in expression over the time points by using a moderated F test [54]. This test is similar to an ANOVA method for each probe except that the residual standard errors are moderated across genes, borrowing information from the ensemble of genes to ensure more stable inference for each gene. One of the advantages of this method is that a gene is not judged as differentially expressed with a very small fold change just because of a small residual standard deviation. The linear models allow for general changes in gene expression between successive time points. The use of dye-swaps in the experimental design allowed a dye-effect to be estimated for each probe. The removal of this technological artifact increased the precision with which differential expression could be detected. The computed *P *values were adjusted for multiple testing by using the Benjamini and Hochberg method to control the false discovery rate (FDR) [55]. Genes were considered to be significant if the adjusted *P *values were < 0.01 (expected FDR no more than 1%).

### q PCR

qPCR reactions were performed as described by Callahan *et al*. [39]. Briefly, each reaction was run in triplicate using 50 ng of RNA in a 15 μl reaction volume using the Superscript III Platinum SYBR Green qRT-PCR Kit (Invitrogen). Primer sequences were designed from available peach and apple ESTs sequences (Additional File 5). The reactions were performed on a 7900 DNA Sequence detector (Applied Biosystems, CA, USA). Quantification was performed using a relative curve derived from a standard RNA run in parallel with each primer pair. A primer set designed to amplify 26S ribosomal RNA [39] was run on all samples and used to normalize the data. A dissociation curve was run to verify that a single desired amplified product was obtained from each reaction.

### Clustering and data mining

Hierarchical clustering was performed using the stats package for R utilizing the Euclidean distance for computing the distance matrix and the complete method for the agglomeration. Data was scaled to a maximum expression of 1 before clustering in order to identify similar patterns of expression. Clusters were plotted in SPlus v 6.0 (Insightful, Washington, USA). KMC, FOM and PTM were performed using the TM4 package version 4.3.01 [27]. In all cases default statistical parameters were used. A cluster value of 12 was chosen for KMC analysis based on the change in slope. A threshold *P*-value of 0.01 was used for PTM.

Gene expression data for apple fruit development was obtained from Janssen *et al*. [29]. Tomato and pepper expression data were obtained from the Tomato Functional Genomics Database [28,20]. Genes matching PP, lignin and flavonoid pathway genes were identified via BlastX using a complete set of pathway genes obtained from The Arabidopsis Information Resource [56]. All expression data were normalized to the youngest fruit sample in each series. Centroid graphs for each dataset were created using the TM4 package version 4.3.01 [27].

### Data Deposition

All microarray data was deposited in the Genome Database for Rosaceae http://www.rosaceae.org/groups/dardick/.

## Abbreviations

4CL: 4-coumarate CoA ligase; ALC: *ALCATRAZ*; C3H: *p*-coumarate 3-hydroxylase; C4H: cinnamate 4-hydroxylase; CCoAOMT: caffeoyl-CoA O-methyltransferase; CCR: cinnamoyl CoA reductase; CHS: chalcone synthase; COMT: catechol-O-methyl transferase; DAB: days after bloom; DFR: dihydroflavonol 4-reductase; F3H: flavanone-3-hydroxylase; FDR: false discovery rate; FOM: Figure of merit; FUL: *FRUITFULL*; IND: *INDEHISCENT*; KMC: K-means clustering; LACC: Laccase; LDOX: leucoanthocyanidin dioxygen-ase; NAC: NAM, ATAF and CUC transcription factors; NST: secondary wall thickening promoting factor; PAL: phenylalanine ammonia-lyase; PCR: polymerase chain reaction; POX: peroxidase; PP: phenylpropanoid pathway; PTM: Pavlidis template matching; qPCR: quantitative PCR; RPL: *REPLUMLESS*; SHP: *SHATTERPROOF*; STK: *SEEDSTICK*; TF: transcription factor.

## Authors' contributions

CDD and AMC contributed equally to this work. CDC and AMC participated in the design, execution, and analyses of the study. RC carried out the array studies and analysis of the data. RJS oversaw the statistical design of the analyses and the apple array studies and the analyses of all the microarray data. MCP provided oversight to the design and data analyses. RS participated in the design and provided oversight to the data analyses. All authors critically read and improved earlier drafts and approved the final manuscript.

## Supplementary Material

Additional file 1 **Microarray experimental design**. Total RNA derived from fruit collected at seven developmental time points were labelled and hybridized to a 15K apple array or a 5K peach array. A reference design was used where each labelled cDNA sample was co-hybridized with labelled RNA from an 87 days after bloom (DAB) reference sample that had the stone removed. For the peach array studies, only four time points were included (37, 40, 45 and 51 DAB). Each time point was represented by three biological samples (>5 fruit from three trees each) and a dye swap was used for each yielding 24 combinations for the peach arrays and 42 combinations for the apple arrays. Click here for file

Additional file 2 Statistically significant genes derived from peach and apple microarray studies. Click here for file

Additional file 3 **Correlation between peach and apple microarray results**. Graphs show expression data from both the peach (red circles) and apple (solid lines) array platforms for a random set of 25 shared genes. Y-axis values are Log_2_-fold change. X-axis values are days after bloom. Click here for file

Additional file 4 K-means clustering data for the combined peach and apple microarray data. Click here for file

Additional file 5 List of primers sequences and gene accession numbers used for quantitative polymerase chain reaction studies. Click here for file

Additional file 6 **Division of phenylpropanoid pathway, lignin and flavonoid gene expression patterns**. Graphs showing absolute expression values obtained from normalized quantitative polymerase chain reaction data. Data for each gene was plotted in a linear curve. *Y*-axis represents normalized relative expression value. *X*-axis is days after bloom (DAB). Corresponding pathway is indicated after each gene abbreviation as phenylpropanoid _*P*_, lignin _*L *_or flavonoid _*F*_. Graphs were grouped into two classes; those with a dominant peak at 47 DAB (left) and those with multiple peaks at 30, 40 and/or 47 DAB (right). Peaks are highlighted by vertical dotted lines. Click here for file

Additional file 7 **Endocarp specific expression of some phenylpropanoid (PP) and lignin pathway genes**. Graph shows expression for 11 PP, lignin and flavonoid pathway genes in dissected fruit harvested at 47 days after bloom. Quantitative polymerase chain reaction data were converted to percent expression relative to the sum total of the dissected parts (*Y*-axis). Genes showing stone specific expression are indicated with an asterix. Click here for file

Additional file 8 **Comparison of phenylpropanoid and lignin gene expression in stone, 87 days after bloom reference and developing wood**. Wood RNA samples were collected from 2-year-old peach stems with the bark removed. Bar graph shows normalized relative expression values (*Y*-axis) derived from quantitative polymerase chain reaction based on a standard dilution curve. Click here for file

Additional file 9 Identification of lignin and flavonoid specific regulons using Pavlidis template matching. Click here for file

Additional file 10 **Lignin and flavonoid pathway induction in early fruit**. Microarray expression data from peach, apple, tomato and pepper (indicated on left) were mined for flavonoid and lignin pathway genes (indicated on top). Centroid graphs show the overall expression pattern for the entire set of genes for each pathway. *X*-axis values are Log_2_-fold change. *Y*-axis is in days after flowering (peach), days after anthesis (apple) and days after pollination (tomato and pepper). The early development stages (prior to ripening) are shown in white while the ripening stages are highlighted gray. Click here for file

Additional file 11 Expression of lignin and flavonoid pathway genes in developing peach, apple, tomato and pepper fruit. Click here for file

Additional file 12 **Analysis of candidate NAM, ATAF and CUC transcription factors (NAC) and MYB regulatory transcription factors identified from the microarray data**. Quantitative polymerase chain reaction results are shown for two NAC class and three MYB class transcription factors in each tissue section: endocarp (red), mesocarp (blue), exocarp (green). Relative expression values are graphed for each tissue section (mesocarp, endocarp and exocarp). *Y*-axis is relative expression value. *X*-axis values are in days after bloom. Click here for file
