# Supplementary figures and images for: Stone formation in peach fruit exhibits spatial coordination of the lignin and flavonoid pathways and similarity to Arabidopsis dehiscence
Source: BMC Biol. 2010 Feb 9;8:13. doi: 10.1186/1741-7007-8-13 (PMC2830173; doi:10.1186/1741-7007-8-13)

## Slide 1
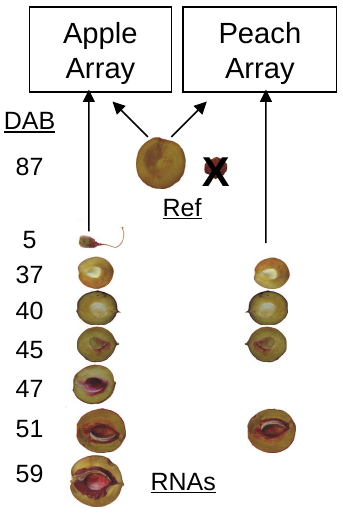

Apple Array
Peach Array
DAB
87
5
37
40
45
47
51
59
X
Ref
RNAs

Supplement: Additional file 1 — Microarray experimental design. Total RNA derived from fruit collected at seven developmental time points were labelled and hybridized to a 15K apple array or a 5K peach array. A reference design was used where each labelled cDNA sample was co-hybridized with labelled RNA from an 87 days after bloom (DAB) reference sample that had the stone removed. For the peach array studies, only four time points were included (37, 40, 45 and 51 DAB). Each time point was represented by three biological samples (>5 fruit from three trees each) and a dye swap was used for each yielding 24 combinations for the peach arrays and 42 combinations for the apple arrays. [file 1741-7007-8-13-S1.PPT]

## Slide 1
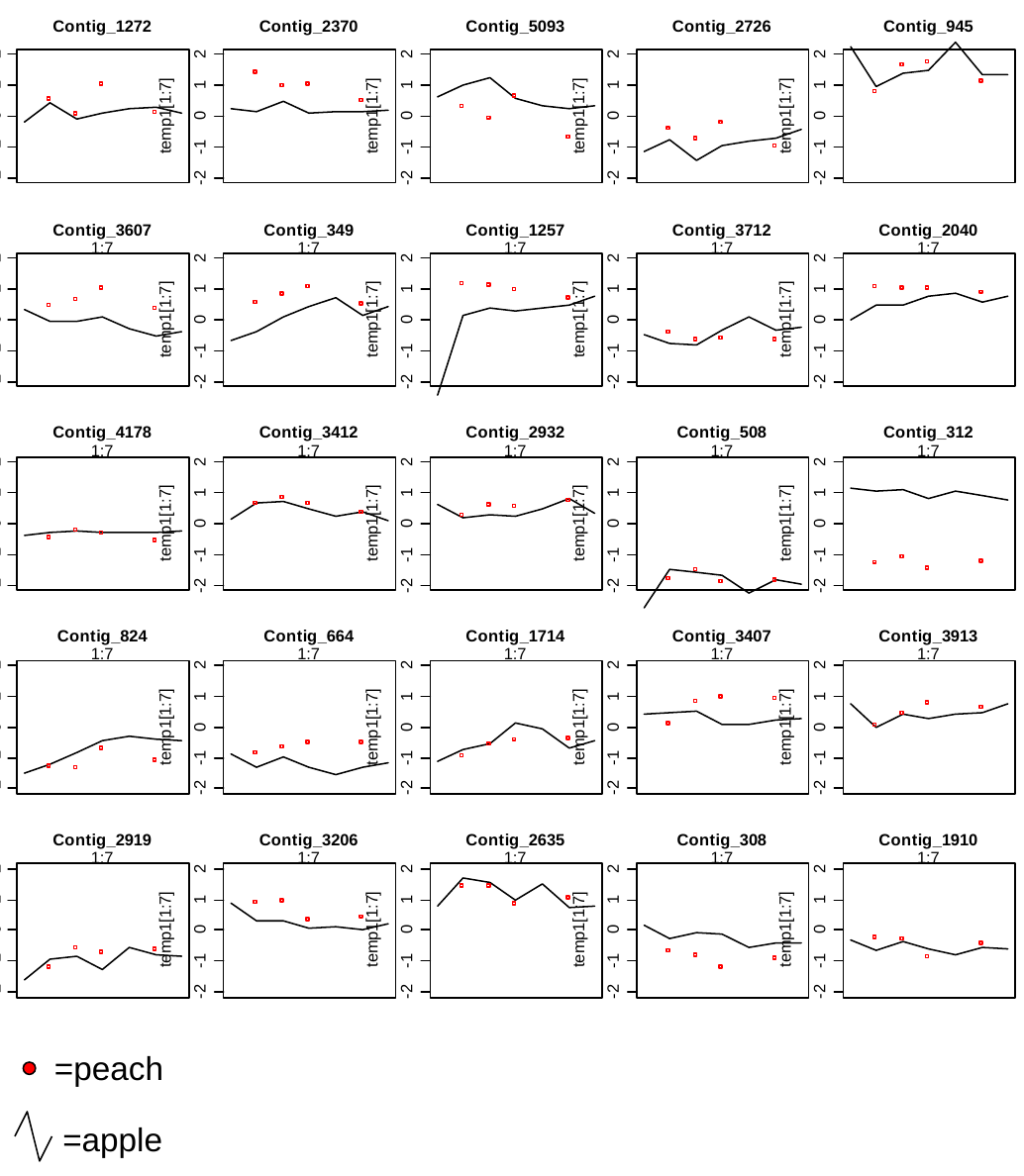

=peach
=apple

Supplement: Additional file 3 — Correlation between peach and apple microarray results. Graphs show expression data from both the peach (red circles) and apple (solid lines) array platforms for a random set of 25 shared genes. Y-axis values are Log2-fold change. X-axis values are days after bloom. [file 1741-7007-8-13-S3.PPT]

## Slide 1
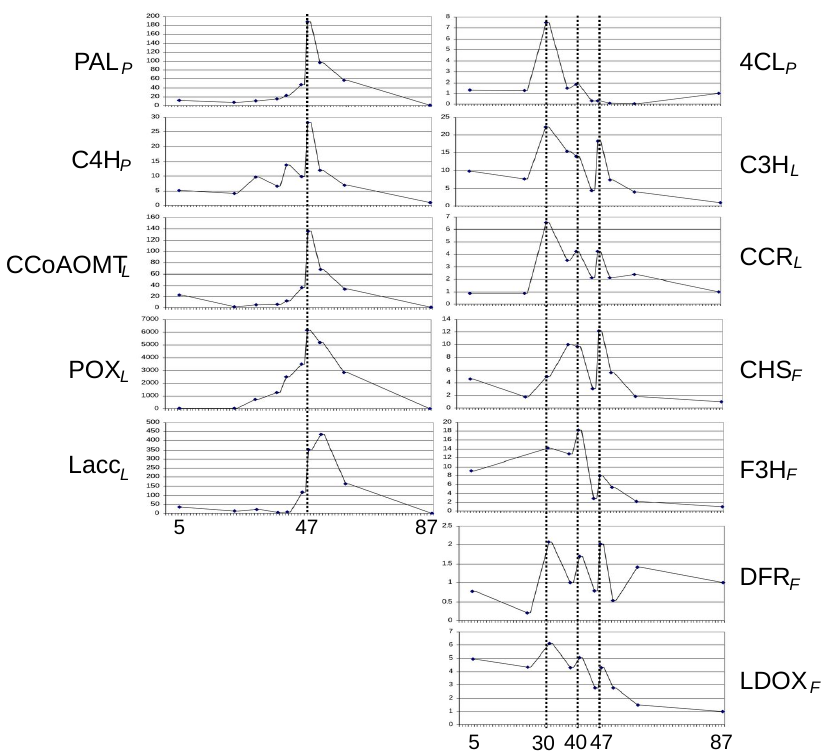

4CL
PAL
P
P
C4H
C3H
P
L
CCR
CCoAOMT
L
L
POX
CHS
F
L
Lacc
F3H
L
F
5
47
87
DFR
F
LDOX
F
5
40
47
87
30

Supplement: Additional file 6 — Division of phenylpropanoid pathway, lignin and flavonoid gene expression patterns. Graphs showing absolute expression values obtained from normalized quantitative polymerase chain reaction data. Data for each gene was plotted in a linear curve. Y-axis represents normalized relative expression value. X-axis is days after bloom (DAB). Corresponding pathway is indicated after each gene abbreviation as phenylpropanoid P, lignin L or flavonoid F. Graphs were grouped into two classes; those with a dominant peak at 47 DAB (left) and those with multiple peaks at 30, 40 and/or 47 DAB (right). Peaks are highlighted by vertical dotted lines. [file 1741-7007-8-13-S6.PPT]

## Slide 1
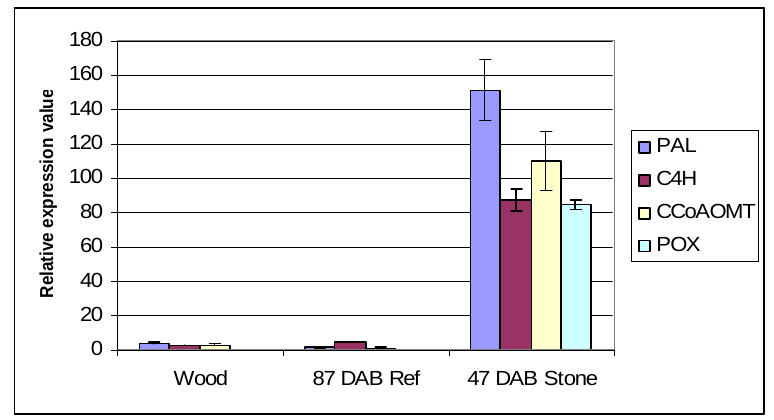

Supplement: Additional file 8 — Comparison of phenylpropanoid and lignin gene expression in stone, 87 days after bloom reference and developing wood. Wood RNA samples were collected from 2-year-old peach stems with the bark removed. Bar graph shows normalized relative expression values (Y-axis) derived from quantitative polymerase chain reaction based on a standard dilution curve. [file 1741-7007-8-13-S8.PPT]

## Slide 1
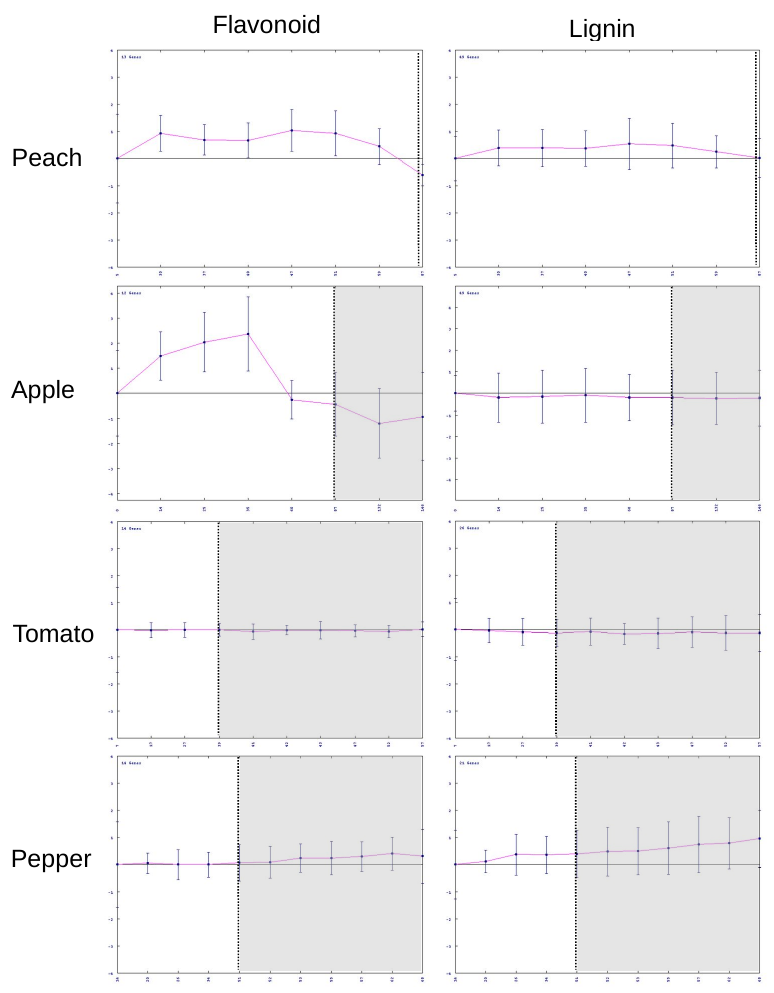

Flavonoid
Lignin
Peach
Apple
Tomato
Pepper

Supplement: Additional file 10 — Lignin and flavonoid pathway induction in early fruit. Microarray expression data from peach, apple, tomato and pepper (indicated on left) were mined for flavonoid and lignin pathway genes (indicated on top). Centroid graphs show the overall expression pattern for the entire set of genes for each pathway. X-axis values are Log2-fold change. Y-axis is in days after flowering (peach), days after anthesis (apple) and days after pollination (tomato and pepper). The early development stages (prior to ripening) are shown in white while the ripening stages are highlighted gray. [file 1741-7007-8-13-S10.PPT]

## Slide 1
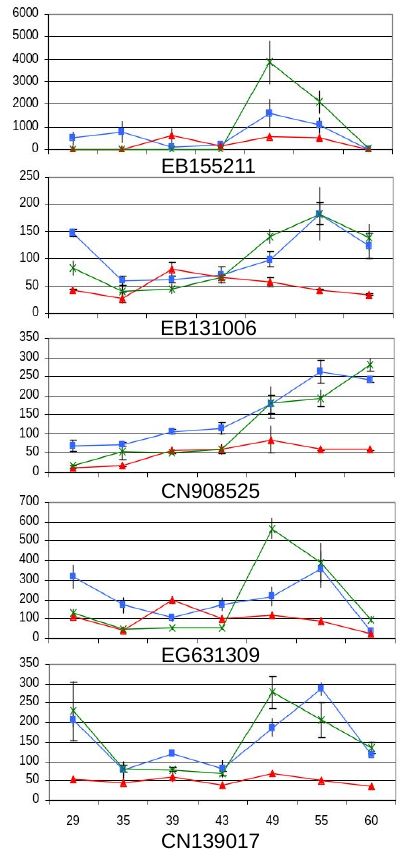

EB155211
EB131006
CN908525
EG631309
CN139017

Supplement: Additional file 12 — Analysis of candidate NAM, ATAF and CUC transcription factors (NAC) and MYB regulatory transcription factors identified from the microarray data. Quantitative polymerase chain reaction results are shown for two NAC class and three MYB class transcription factors in each tissue section: endocarp (red), mesocarp (blue), exocarp (green). Relative expression values are graphed for each tissue section (mesocarp, endocarp and exocarp). Y-axis is relative expression value. X-axis values are in days after bloom. [file 1741-7007-8-13-S12.PPT]
